# Improved Bacterial 16S rRNA Gene (V4 and V4-5) and Fungal Internal Transcribed Spacer Marker Gene Primers for Microbial Community Surveys

**DOI:** 10.1128/mSystems.00009-15

**Published:** 2015-12-22

**Authors:** William Walters, Embriette R. Hyde, Donna Berg-Lyons, Gail Ackermann, Greg Humphrey, Alma Parada, Jack A. Gilbert, Janet K. Jansson, J. Gregory Caporaso, Jed A. Fuhrman, Amy Apprill, Rob Knight

**Affiliations:** a Department of Molecular Biology and Genetics, Cornell University, Ithaca, New York, USA; b Department of Pediatrics, University of California at San Diego, La Jolla, California, USA; c BioFrontiers Institute, University of Colorado at Boulder, Boulder, Colorado, USA; d Department of Biological Sciences, University of Southern California, Los Angeles, California, USA; e Biosciences Division (BIO), Argonne National Laboratory, Argonne, Illinois, USA; f Departments of Ecology and Evolution and Surgery, The University of Chicago, Chicago, Illinois, USA; g Institute for Genomic and Systems Biology, The University of Chicago, Chicago, Illinois, USA; h The Marine Biological Laboratory, Woods Hole, Massachusetts, USA; i The Field Museum of Natural History, Chicago, Illinois, USA; j Earth and Biological Sciences Directorate, Pacific Northwest National Laboratory, Richland, Washington, USA; k Department of Biological Sciences, Northern Arizona University, Flagstaff, Arizona, USA; l Department of Marine Chemistry and Geochemistry, Woods Hole Oceanographic Institution, Woods Hole, Massachusetts, USA; m Department of Computer Science and Engineering Department, University of California at San Diego, La Jolla, California, USA; University of Birmingham

**Keywords:** microbial ecology, marker genes, primers, 16S, ITS

## Abstract

Designing primers for PCR-based taxonomic surveys that amplify a broad range of phylotypes in varied community samples is a difficult challenge, and the comparability of data sets amplified with varied primers requires attention. Here, we examined the performance of modified 16S rRNA gene and internal transcribed spacer (ITS) primers for archaea/bacteria and fungi, respectively, with nonaquatic samples. We moved primer bar codes to the 5′ end, allowing for a range of different 3′ primer pairings, such as the 515f/926r primer pair, which amplifies variable regions 4 and 5 of the 16S rRNA gene. We additionally demonstrated that modifications to the 515f/806r (variable region 4) 16S primer pair, which improves detection of *Thaumarchaeota* and clade SAR11 in marine samples, do not degrade performance on taxa already amplified effectively by the original primer set. Alterations to the fungal ITS primers did result in differential but overall improved performance compared to the original primers. In both cases, the improved primers should be widely adopted for amplicon studies.

**IMPORTANCE** We continue to uncover a wealth of information connecting microbes in important ways to human and environmental ecology. As our scientific knowledge and technical abilities improve, the tools used for microbiome surveys can be modified to improve the accuracy of our techniques, ensuring that we can continue to identify groundbreaking connections between microbes and the ecosystems they populate, from ice caps to the human body. It is important to confirm that modifications to these tools do not cause new, detrimental biases that would inhibit the field rather than continue to move it forward. We therefore demonstrated that two recently modified primer pairs that target taxonomically discriminatory regions of bacterial and fungal genomic DNA do not introduce new biases when used on a variety of sample types, from soil to human skin. This confirms the utility of these primers for maintaining currently recommended microbiome research techniques as the state of the art.

## Abstract (precis)

We continue to uncover a wealth of information connecting microbes in important ways to human and environmental ecology. As our scientific knowledge and technical abilities improve, the tools used for microbiome surveys can be modified to improve the accuracy of our techniques, ensuring that we can continue to identify groundbreaking connections between microbes and the ecosystems they populate, from ice caps to the human body. It is important to confirm that modifications to these tools do not cause new, detrimental biases that would inhibit the field rather than continue to move it forward. We therefore demonstrated that two recently modified primer pairs that target taxonomically discriminatory regions of bacterial and fungal genomic DNA do not introduce new biases when used on a variety of sample types, from soil to human skin. This confirms the utility of these primers for maintaining currently recommended microbiome research techniques as the state of the art.

## INTRODUCTION

Surveys of the small ribosomal subunits (SSUs) for *Bacteria*, *Archaea*, and *Eukarya* and the internal transcribed spacer (ITS) region for fungi are central to microbial ecology. Multiple primer pairs are available for each of these marker genes, each pair associated with its own taxon biases. Marker gene databases are frequently updated, and the updated information can include new microbial lineages with suboptimal or poor binding to existing PCR primers; to maximize taxonomic sensitivity in light of these new data, primers may need to be periodically redesigned. Accordingly, we tested modified forms of the 515f and 806r 16S rRNA gene (variable region 4) primers (1), introduced a bar-coding scheme to allow alternative reverse primer pairing along with testing a construct spanning variable region V4-5 (515f/926r), which was compared to the V4 construct, and we compared alternative ITS1-spanning constructs for fungal analysis.

The 515f-806r bacterial/archaeal primer pair, traditionally used by the Earth Microbiome Project (EMP; http://www.earthmicrobiome.org/emp-standard-protocols/16s/), was recently shown to be biased against both the *Crenarchaeota*/*Thaumarchaeota* (2), important environmental archaea, and the SAR11 clade, which is abundant in aquatic bacteria (3). Parada et al. (4) and Apprill et al. (5) have modified the 515f/806r 16S rRNA gene primer pair first designed for use with the Illumina platform, as described by Caporaso et al. in 2011 (1), to reduce these biases. Specifically, additional degeneracy was added to the 515f primer to reduce bias against *Crenarchaeota*/*Thaumarchaeota* (4) and to the 806r primer to minimize the bias against the SAR11 clade (5). Original and new PCR primer sequences are listed in Table 1. For fungal surveys, the ITS1F (6) and ITS2 primer pair (7) has been commonly used. This primer pair has also recently been modified by Smith and Peay (8), who used the original ITS1F/ITS2 primers (6, 7) but designed sequencing primers that extended into the amplicon region to provide greater specificity for ITS1. All PCR and sequencing primer constructs, including the ITS sequencing primer modifications made by Smith and Peay, are detailed in Table S1 in the supplemental material.

**TABLE 1:** PCR primer sequences used in this study (old and new constructs)

Primer name	Primer sequence^a^	Reference
515f Original	GTGCCAGCMGCCGCGGTAA	Caporaso et al. (1)
806r Original	GGACTACHVGGGTWTCTAAT	Caporaso et al. (1)
515f Modified	GTG**Y**CAGCMGCCGCGGTAA	Parada et al. (4)
806r Modified	GGACTAC**N**VGGGTWTCTAAT	Apprill et al. (5)
926r	CCGYCAATTYMTTTRAGTTT	Parada et al. (4)
ITS1f	CTTGGTCATTTAGAGGAAGTAA	Gardes and Bruns (6)
ITS2	GCTGCGTTCTTCATCGATGC	White et al. (7)

^a^ Primers are listed in a 5′-to-3′ orientation. The original 515f and 806r primers are listed for comparison to the added degeneracy in the new constructs (changes are shown in bold). See Smith and Peay (8) for further details on the modifications to the ITS sequencing primers.

10.1128/mSystems.00009-15.1 Table S1  All Golay barcoded 515f/806r, 515f/926r, and ITS PCR primer and sequencing constructs. Download Table S1, XLSX file, 6.1 MB. Copyright © 2015 Walters et al. 2015 Walters et al. This content is distributed under the terms of the Creative Commons Attribution 4.0 International license.

An important question is whether or not the modifications to these primers correct the known biases against specific microbial taxa without introducing detrimental biases compared to data produced using the original primer pairs. Indeed, the new constructs produce data comparable to those produced with the old constructs. By selecting studies representative of a wide range of sample types, from stool and soil to the built environment, we compared the new and old constructs for both 16S rRNA and ITS marker genes, and we also compared the performance of the modified 515f/806rB (V4) primer constructs to the longer 515f/926r (V4-5) constructs.

## RESULTS AND DISCUSSION

### Assessing the performance of the new primer constructs compared to the old constructs by using nonmarine, vertebrate-associated, and environment-associated microbial communities.

To confirm that the modified primer pairs did not introduce new, detrimental biases while correcting known biases against particular taxa, we tested the performance of the old, unmodified primer constructs to the performance of the new, modified primer constructs using a sample set not expected to contain large amounts of clade SAR11 or *Thaumarchaeota*/*Crenarchaeota*.

Bacterial 16S V4 region sequences were processed using QIIME 1.8.0 (9). Default parameters were used for demultiplexing and quality filtering of reads. After quality filtering, the data set amplified with the original 515f/806r constructs had a total of 2,896,510 reads, and the data set amplified with the 515f/806rB constructs had a total of 7,646,323 reads. The difference in read number was not related to a difference in primer pair performance, as the amplicons produced with the original primer construct were sequenced together with those from another unrelated study, whereas the amplicons produced with the modified primer constructs were not sequenced with another study. Closed-reference operational taxonomic unit (OTU) picking was performed on sequences generated with the old and new primer constructs together by clustering against information in the August 2013 release of the Greengenes database (10), with approximately 73.6% of the reads clustering at 97% identity. The sequences were also clustered closed-reference against the SILVA 111 release for the purpose of comparing the performance of the 515f/806r and 515f/926r primer constructs, as the 515f/926r pair is expected to amplify eukaryotes, which are represented in the SILVA database.

Fungal ITS1-spanning sequences were also processed using QIIME 1.8.0 (9) and quality filtered using default parameters. After quality filtering, the number of reads associated with the data set amplified with the original ITS constructs was 11,105,808, while the number of reads associated with the modified constructs was 11,655,576. Sequences produced from both the old and modified ITS primers were clustered closed-reference against the UNITE May 2014 release of the Dynamic Developer database, with ~23% of the original ITS1 constructs clustering at 97% identity and ~50.1% of modified constructs clustering. Reads could fail to cluster due to poor representation of fungi represented in the query reads or due to the query reads being off-target sequences. With a lower clustering percent identity of 90%, ~35% of the original ITS1 compared to ~79% of the modified constructs clustered successfully, indicating that the new ITS1 constructs have improved specificity for ITS reads.

**FIG 1:**
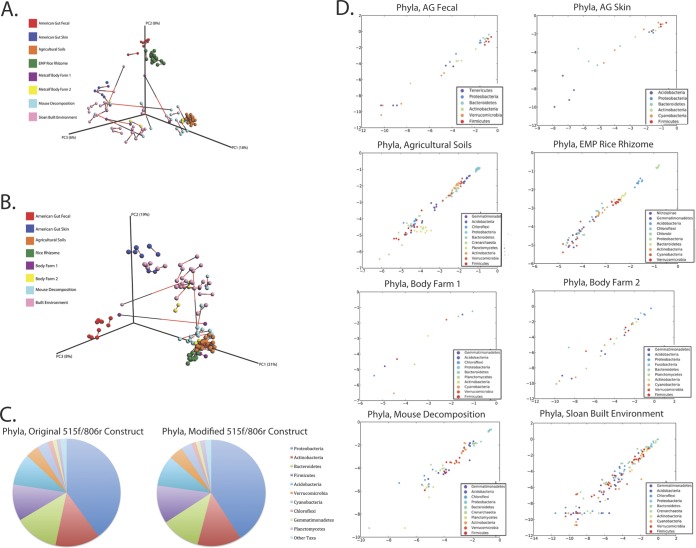
Comparison of the original 515f/806r primer pair and the new, modified 515f/806rB primer pair. (A) Procrustes plot of original and modified 515f/806r constructs, with unweighted UniFrac metric, M*^2^* = 0.111. (B) Original and modified 515f/806r constructs, weighted UniFrac metric *M*^2^ = 0.196. (C) Pie charts illustrating the mean relative abundance of phyla present (all studies combined) in samples amplified with the old 515f/806r construct or with the modified 515f/806rB construct. (D) Taxa scatterplots for the original and modified 515f/806r primers. Phyla plots are shown for American Gut fecal, American Gut skin, agricultural soils, EMP Rice Rhizome, Body Farm 1, Body Farm 2, mouse decomposition, and Sloan built environment samples. Outlier samples have been removed from the results shown.

Procrustes plots comparing the UniFrac distance matrices of samples amplified using the original 515/806r primer construct and the new 515f/806r construct (Fig. 1A and B) revealed that most samples produced highly comparable results (*M*^2^ = 0.111, *P* < 0.05, and Mantel *r* statistic = 0.897, *P* = 0.01 for the unweighted UniFrac-based plot; *M*^2^ = 0.196, *P* < 0.05, and Mantel *r* statistic = 0.909, *P* = 0.01 for the weighted UniFrac-based plot). The relative abundances of the most abundant taxa present were also comparable between the 515f/806r and modified 515f/806rB primer constructs (Fig. 1C). Scatterplots (Fig. 1D; see also Fig. S2 in the supplemental material) agree with these results, revealing a strong concordance between the old and new 515f/806r primer constructs (Table 2; see also Table S2 in the supplemental material) across levels from phylum to genus.

10.1128/mSystems.00009-15.2 Figure S1  Predicted amplified taxa for modified 515f/806r and 515f/926r primer pairs. One mismatch was allowed for either primer in any position except for the last five 3′ bases of the primers. (A) 515f/806r archaeal taxa. (B) 515f/806r eukaryotic taxa. (C) 515f/806r bacterial taxa. (D) 515f/926r archaeal taxa. (E) 515f/926r eukaryotic taxa. (F) 515f/926r bacterial taxa. Taxa are listed in order of sequence abundance in the SILVA 111 reference database, from left to right (for panels A, B, D, and E) or from top to bottom (panels C and F). Download Figure S1, PDF file, 1.3 MB. Copyright © 2015 Walters et al. 2015 Walters et al. This content is distributed under the terms of the Creative Commons Attribution 4.0 International license.

10.1128/mSystems.00009-15.3 Figure S2  Taxa scatterplots for the original and modified 515f/806r primers. Phyla, class, order, family, and genus plots are shown for American Gut fecal, American Gut skin, agricultural soils, EMP Rice Rhizome, Body Farm 1, Body Farm 2, mouse decomposition, and Sloan built environment samples. Download Figure S2, PDF file, 2.8 MB. Copyright © 2015 Walters et al. 2015 Walters et al. This content is distributed under the terms of the Creative Commons Attribution 4.0 International license.

**TABLE 2:** Relationship between the original and modified 515f/806r primer pair taxonomy abundances

Taxonomic level	*R*^2^ value for sample source^a^							
	AG fecal	AG skin	Agricultural soil	Rice Rhizome	Body Farm 1	Body Farm 2	Mouse decomposition	Sloan built environment
Phylum	0.9780	0.8833	0.9546	0.9799	0.8630	0.9172	0.9075	0.9148
Class	0.9434	0.8283	0.8613	0.9398	0.5579	0.8982	0.6658	0.8532
Order	0.8414	0.8928	0.9178	0.8291	0.2961	0.6644	0.6460	0.8148
Family	0.9392	0.7712	0.9270	0.8942	0.6181	0.8454	0.7769	0.8161
Genus	0.9400	0.7914	0.9082	0.8466	0.5092	0.8690	0.6794	0.8033

^a^ AG, Animal Gut study. The Sloan “built environment” was a house.

10.1128/mSystems.00009-15.4 Table S2  Relationship between the original and modified 515f/806r primer pair taxonomy abundance levels (*R*^2^ values for each taxonomic level for each sample type/study [outliers included] are listed). Download Table S2, DOCX file, 15 KB. Copyright © 2015 Walters et al. 2015 Walters et al. This content is distributed under the terms of the Creative Commons Attribution 4.0 International license.

**FIG 2:**
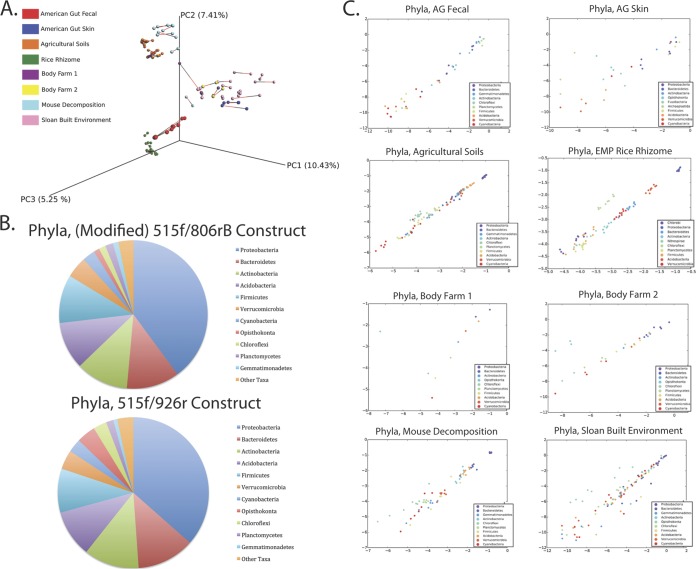
Comparison of the modified 515f/806rB V4 primer pair and the 515f (modified)/926r primer pair. (A) Procrustes plot of modified 515f/806r and 515f/926r constructs, Bray-Curtis dissimilarity *M*^2^ = 0.058. (B) Pie charts illustrating the mean relative abundance of phyla present (all studies combined) in samples amplified with the modified 515f/806rB construct or with the 515f/926r construct. (C) Taxa scatterplots for the modified 515f/806rB construct and the 515f/926r construct. Phyla plots are shown for American Gut fecal, American Gut skin, agricultural soils, EMP Rice Rhizome, Body Farm 1, Body Farm 2, mouse decomposition, and Sloan built environment samples. Outlier samples have not been removed from the data shown.

Comparisons of modified V4 primers and V4-5 constructs revealed strong concordance. Procrustes analysis (Fig. 2A) showed similar clustering patterns (*M*^2^ = 0.058, *P* < 0.05, and Mantel *r* statistic = 0.975; *P* = 0.01 with the Bray-Curtis metric) (Fig. 2A), with especially strong clustering observed among commonly studied sample types (stool, soil, skin). The relative abundances of the most abundant phyla were also comparable between both primer constructs (Fig. 2B), and scatterplots revealed that the concordance between the 515f/806rB construct and the longer 515f/926r construct was strong (Fig. 2C and Table 3; see also Fig. S3 and Table S3 in the supplemental material).

10.1128/mSystems.00009-15.5 Figure S3  Taxa scatterplots for the modified 515f/806r and 515f/926r primers. Phyla, class, order, family, and genus plots are shown for American Gut fecal, American Gut skin, agricultural soils, EMP Rice Rhizome, Body Farm 1, Body Farm 2, mouse decomposition, and Sloan built environment samples. Download Figure S3, PDF file, 2.8 MB. Copyright © 2015 Walters et al. 2015 Walters et al. This content is distributed under the terms of the Creative Commons Attribution 4.0 International license.

**TABLE 3:** Relationships between the modified 515f/806r and 515f/926r primer pair taxonomy abundances

Taxonomic level	*R*^2^ value for sample source^a^							
	AG fecal	AG skin	Agricultural soil	Rice Rhizome	Body Farm 1	Body Farm 2	Mouse decomposition	Sloan built environment
Phylum	0.9733	0.6540	0.9743	0.9156	0.2373	0.6763	0.9232	0.8315
Class	0.9543	0.5998	0.9398	0.7284	0.7276	0.9643	0.9155	0.7597
Order	0.9653	0.5027	0.9005	0.2997	0.8705	0.9578	0.9124	0.7949
Family	0.9528	0.6028	0.8808	0.4555	0.7772	0.9417	0.7478	0.7919
Genus	0.8841	0.6525	0.8476	0.3569	0.6867	0.9271	0.6458	0.8084

^a^ AG, Animal Gut study. The Sloan “built environment” was a house.

10.1128/mSystems.00009-15.6 Table S3  Relationship between the modified 515f/806r and 515f/926r primer pair taxonomy abundance levels (*R*^2^ values for each taxonomic level for each sample type/study [outliers included] are listed). Download Table S3, DOCX file, 15 KB. Copyright © 2015 Walters et al. 2015 Walters et al. This content is distributed under the terms of the Creative Commons Attribution 4.0 International license.

Four soil sample pairs (a total of 8 samples out of 86 samples tested; samples 3.7.13A, 3.8.13.B, D.soil.T0.1, and F.CTRL_soil.T0.4) from the human and mouse decomposition data sets did not perform comparably between the 515f/806r and 515f/806rB or 515f/806r and 515f/926r constructs; however, the taxonomic compositions of these samples were not typical of soil. We hypothesize that the dissimilarities were not due to differences in primer constructs but may have been due to sample DNA depletion that occurred as a result of testing multiple primer constructs, thus leading to spurious amplifications during PCR; however, we cannot confirm this hypothesis with the data presently available. Additionally, further analyses, such as with mock communities (4), could evaluate the extent to which differences in these outliers stem from systematic biases of particular taxa. These outliers were removed from the analyses depicted in Tables 2 and 3 and also Fig. S2 and S3 in the supplemental material, but they were retained for the analyses depicted in Tables S2 and S3 in the supplemental material.

**FIG 3:**
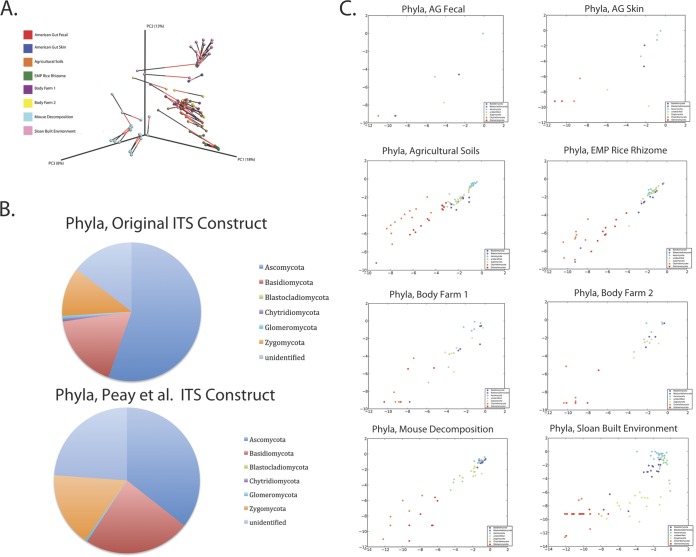
Comparison of the original ITS primer pair and the new, modified ITS primer pair. (A) Procrustes plot of original and modified ITS1 constructs, Bray-Curtis dissimilarity *M*^2^ = 0.363. (B) Pie charts illustrating the mean relative abundance of phyla present (all studies combined) in samples amplified with the old ITS construct and the modified ITS construct. (C) Taxa scatterplots for the original and modified ITS primers. Phyla plots are shown for American Gut fecal, American Gut skin, agricultural soils, EMP Rice Rhizome, Body Farm 1, Body Farm 2, mouse decomposition, and Sloan built environment samples. Outlier samples have been removed from the data shown.

The modifications to the fungal ITS sequencing primers approximately doubled the yield of reads that clustered against the reference ITS database. Unlike the modified 16S primers, the taxonomies generated by these primers have sizable differences. Procrustes analysis revealed a moderate concordance between the original and modified ITS primer constructs (*M*^2^ = 0.363, *P* < 0.05, and Mantel *r* statistic = 0.877, *P* = 0.01 with the Bray-Curtis metric) (Fig. 3A). Study-specific differences were observed; the clustering patterns of rice rhizome and agricultural soil samples were strongly matched between the two primer constructs, and the clustering pattern of the built environmental samples was moderately well matched; however, the clustering patterns of the American Gut Project samples (skin) and decomposition samples were poorly matched. Scatterplots confirmed the observations obtained from the Procrustes analysis (Fig. 3B and C; see also Fig. S4 in the supplemental material), indicating strong concordance between the old and new ITS primer constructs for the soil and rice rhizome data sets but weaker concordance for the other data sets with the poorest concordances observed among the American Gut skin and decomposition samples. *R*^2^ values varied widely from study to study (Table 4). Notably, the modified primers detected less *Ascomycetes* and more of all other fungal taxa. The likely explanation for the observed differences between the two primer constructs is the increased number of reads that successfully clustered against the UNITE database from amplicons produced by the new constructs. This is particularly important given that data sets expected to have a large fungal community performed similarly using both primer constructs, but data sets that may be expected to have low fungal presence and/or diversity were more affected by increased clustering against the UNITE database, suggesting that when performing closed-reference OTU picking, the new primer pair is more useful for fungal community analyses on these data types by providing increased specificity for fungal ITS reads.

10.1128/mSystems.00009-15.7 Figure S4  Taxa scatterplots for the original and modified (Smith and Peay) fungal ITS primers. Phyla, class, order, family, and genus plots are shown for American Gut fecal, American Gut skin, agricultural soils, EMP Rice Rhizome, Body Farm 1, Body Farm 2, mouse decomposition, and Sloan built environment samples. Download Figure S4, PDF file, 2.7 MB. Copyright © 2015 Walters et al. 2015 Walters et al. This content is distributed under the terms of the Creative Commons Attribution 4.0 International license.

**TABLE 4:** Relationship between the ITS primer pair taxonomy abundances

Taxonomic level	*R*^2^ value for sample source^a^							
	AG fecal	AG skin	Agricultural soil	Rice Rhizome	Body Farm 1	Body Farm 2	Mouse decomposition	Sloan built environment
Phylum	0.8116	0.6305	0.9017	0.9246	0.9018	0.9030	0.9245	0.7647
Class	0.2469	0.2746	0.6466	0.9389	0.5678	0.3587	0.6212	0.5092
Order	0.2045	0.6538	0.6331	0.9334	0.6620	0.5356	0.4103	0.4808
Family	0.1801	0.7219	0.7005	0.8828	0.4942	0.5281	0.4509	0.5918
Genus	0.1747	0.4990	0.7084	0.8884	0.7411	0.3767	0.3560	0.4033

^a^ AG, Animal Gut study. The Sloan “built environment” was a house.

Here, we showed that the 515f/806rB bacterial/archaeal and ITS fungal primer pairs do not introduce detrimental biases compared to the original constructs in nonaquatic environments. The modified 515f/806rB results were highly concordant with the original 515f/806r and 515f/926r results. The forward-barcoded construct allowed for future primer pairs to be used on the Illumina platform as sequencing read length improves. While the modified ITS primer pair does not yield concordant results as the old primer pair does, the overall performance of the modified ITS construct is improved, because they generate more high-quality reads that match the UNITE fungal database. Knowledge of how various primer pairs compare and amplify taxa is important for study design and relating results to prior studies, which we have addressed here for some of the commonly used primers in microbial surveys.

## MATERIALS AND METHODS

### Description of data sets.

To compare the performance of the new 515f/806rB primer constructs to that of the old 515f-806r constructs, we produced amplicons in six different data sets using both primer pairs and sequenced the resulting amplicons (more details are provided below). We chose samples from a varied list of studies in order to demonstrate the performance of the 515f/806rB primer pair and the modified ITS primer pair on a variety of sample types.

The first set of samples included in this study includes five fecal samples and five skin samples from the American Gut project. All 10 samples were sequenced as part of American Gut round two, available from EBI under accession number ERP012803. All five fecal samples amplified successfully with both primer pairs and were used in downstream analyses. Of the five skin samples, four amplified successfully with both primer pairs and were used in downstream analyses. Sample ID numbers for the samples used in this study are listed in Table S4 in the supplemental material.

10.1128/mSystems.00009-15.8 Table S4  Studies and IDs for samples amplified with the primer pairs used in this study. Download Table S4, DOCX file, 15 KB. Copyright © 2015 Walters et al. 2015 Walters et al. This content is distributed under the terms of the Creative Commons Attribution 4.0 International license.

The second set of samples included in this study is part of a study on agricultural soils (11) available from EBI under accession number ERP002214. We selected 12 samples for use in the current study. All 12 samples amplified successfully with both 16S primer pairs and with both ITS primer pairs and were used in downstream analyses. Sample IDs for the samples used in this study are listed in Table S4 in the supplemental material.

The third set of samples included in this study is part of an unpublished EMP study of the rice rhizome. We selected nine samples for use in the current study. All nine samples amplified successfully with both 16S primer pairs and with both ITS primer pairs and were used in downstream analyses. Sample IDs for the samples used in this study are listed in Table S4 in the supplemental material.

The fourth set of samples included in this study is part of a human decomposition study (12) available from EBI under accession numbers ERP012879 and ERP012880. A total of nine soil and cadaver samples from two human cadavers (placed in March [Body Farm 1] and May [Body Farm 2]) were chosen for use in this study. Six samples were amplified successfully with both 16S primer pairs, and all nine samples were amplified successfully with both ITS primer pairs and were used in downstream analyses. Sample IDs for the samples used in this study are listed in Table S4 in the supplemental material.

The fifth set of samples included in this study is part of a mouse decomposition study (13) available from EBI under accession number ERP003929. We selected 15 soil samples (5 desert soils, 5 forest soils, and 5 grassland soils) for use in the current study. Nine samples were amplified successfully with both 16S primer pairs, and a different set of nine samples were amplified successfully with both ITS primer pairs and were used in downstream analyses. Sample IDs for the samples used in this study are listed in Table S4 in the supplemental material.

The sixth set of samples included in this study is part of a Sloan-funded built environment study (unpublished data), in which various locations within houses were swabbed. We selected 33 samples for use in the current study. A total of 16 samples were amplified successfully with both 16S primer pairs, and a total of 17 samples were amplified successfully with both ITS primer pairs and were used in downstream analyses. Sample IDs for the samples used in this study are listed in Table S4 in the supplemental material.

Mapping files, raw data, and quality-filtered sequences for all of the above studies are available at http://qiita.ucsd.edu under study ID number 10218.

### Microbial DNA extraction, 16S and ITS amplicon production, and amplicon sequencing.

Samples were stored at −80°C as soon as possible after collection until microbial DNA extraction. Microbial genomic DNA was extracted using the PowerSoil DNA isolation kit (MoBio, Carslbad, CA) following Earth Microbiome Project benchmarked protocols. The 16S rRNA V4 amplicons and ITS1-spanning amplicons were both generated using the following Earth Microbiome Project benchmarked protocols (http://www.earthmicrobiome.org/emp-standard-protocols/).

The complete reagent mixture contained PCR-grade water (13 μl), 5′ Hot master mix (10.0 μl), forward primer (10 μM, 0.5 μl), reverse primer (10 μM, 1.0 μl), and template DNA (1.0 μl) in a total reaction volume of 25 μl. The PCR amplification conditions were as follows (384-well thermocycler): 94°C for 3 min; 35 cycles of 94°C for 45 s, 50°C for 60 s, and 72°C for 90s; 72°C for 10 min, and then a 4°C hold. Resulting amplicons were cleaned, pooled, and quantified using the Quant-iT picogreen double-stranded DNA assay kit following EMP benchmarked protocols (http://www.earthmicrobiome.org/emp-standard-protocols/). Pooled amplicons were then sequenced on a multiplexed 2- by 150-bp Illumina MiSeq sequencing run at the BioFrontiers Next Generation Sequencing Facility at the University of Colorado, Boulder.

### Modifications to PCR and sequencing primers.

We first modified the 515f/806r PCR primer construct by bar coding the forward primer, rather than the reverse primer, with 960 unique, 12-base Golay bar codes (14). The addition of bar codes to the forward primer enables the user to produce amplicons spanning multiple 16S rRNA gene variable regions, such as the V4-V5 construct described here. Parada et al. (4) demonstrated improved phylogenetic resolution using the 515f-926r construct; additionally, the percentage of eukaryotic taxa amplified by this construct indicated that in marine or other non-host-associated environments, this construct may be a particularly attractive choice for producing amplicons from all three domains. The forward primer bar-coding schema can also facilitate the amplification of alternative taxa by allowing pairing of the 515f primer with various other reverse primers, taking full advantage of current longer-read Illumina sequencing technology. This approach is advantageous over that of popular dual-bar-coding schemes (15, 16), which do not allow the flexibility that single-ended forward bar coding does, due to the necessity of a variety of bar codes for both the forward and reverse primers.

We added pad regions to increase the melting temperature (*T_m_*) of the sequencing primers to approximately 66°C (calculated using OligoAnalyzer 3.1) based upon Illumina’s technical guidelines for amplicon primers (https://support.illumina.com/content/dam/illumina-support/documents/documentation/chemistry_documentation/16s/16s-metagenomic-library-prep-guide-15044223-b.pdf). The modified PCR and sequencing primers, including the Smith and Peay (2014) modified ITS sequencing primers and the bar-coded primer constructs (see Table S1 in the supplemental material) (http://www.earthmicrobiome.org/emp-standard-protocols/16s/; New Illumina HiSeq 16S primer sequences) were screened for dimers and the secondary structure by using Primer Prospector (17). Each construct was filtered using the check_primer_barcode_dimers script with a score_threshold of −20.0. Predicted taxonomic coverage was generated by scoring each of the primers (analyze_primers script with default settings) against the SILVA 111 97% OTUs database (18), available for download at http://www.arb-silva.de/download/archive/qiime/. Predicted taxonomic coverage for each primer pair was then generated from the scored hits with the taxa_coverage script, allowing a single non-3′-mismatch for the primers with a score_threshold parameter of 0.4. Graphs for overall domain-level and phylum-level taxa were generated from the text output of taxa coverage, which was split by domain and sorted by sequence counts per phylum from the SILVA 111 database (see Fig. S1 in the supplemental material).

### Sequence analysis.

For all sequencing reads, QIIME 1.8.0 was used with default parameters for demultiplexing, quality filtering, and clustering reads into OTUs. Read 1 was used for all comparisons to maintain consistency. The Greengenes, UNITE, and SILVA databases were used to obtain reference sequences and for taxonomy assignment, as described in the Results and Discussion section.

Procrustes analysis was used to assess the comparability of clustering patterns observed in a principal-coordinate analysis (PCoA) space for samples amplified with the old and new primer constructs. Procrustes plots were generated using the transform_coordinate_matrices script on principal coordinate files (generated from unweighted and weighted UniFrac distance matrices for 515f/806r comparisons and Bray-Curtis dissimilarity matrices for ITS data and 515f/806r-versus-515f/926r comparisons) with an even sampling of depth of 1,000 sequences per sample for beta diversity calculations.

Scatterplots were also produced to further assess the concordance between results produced using each pair of primer constructs. First, low-abundance taxa (<0.01%) were filtered from the OTU tables by using the filter_otus_from_otu_table script with the min_count_fraction parameter at 0.0001. Filtered OTU tables were then split according to sample type using the split_otu_table script, and the resulting OTU tables were summarized into taxonomic levels (phylum through genus) via the summarize_taxa script. Scatterplots of log-transformed abundance values and *R*^2^ values were generated from the OTU tables by using the generate_taxa_scatter_plots script (using an *n* of 10 to process the top 10 taxa); this information is located on the following gisthub (https://gist.github.com/walterst/df5826d684babad226d6/download#).
