# Supplementary figures and images for: Improved Bacterial 16S rRNA Gene (V4 and V4-5) and Fungal Internal Transcribed Spacer Marker Gene Primers for Microbial Community Surveys
Source: mSystems. 2015 Dec 22;1(1):e00009-15. doi: 10.1128/mSystems.00009-15 (PMC5069754; doi:10.1128/mSystems.00009-15)

**A**

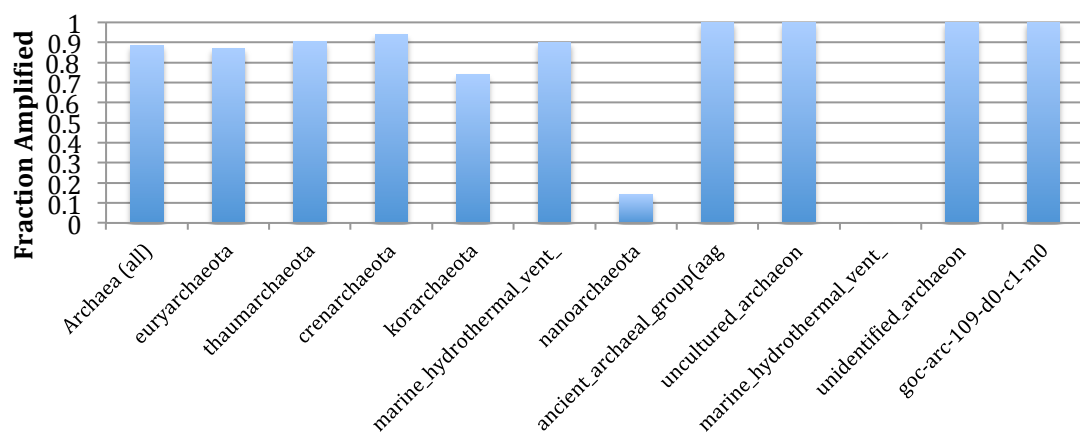

**B**

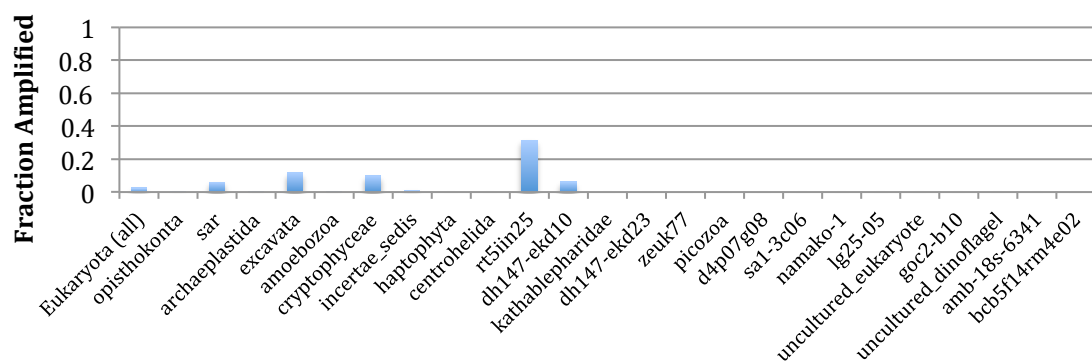

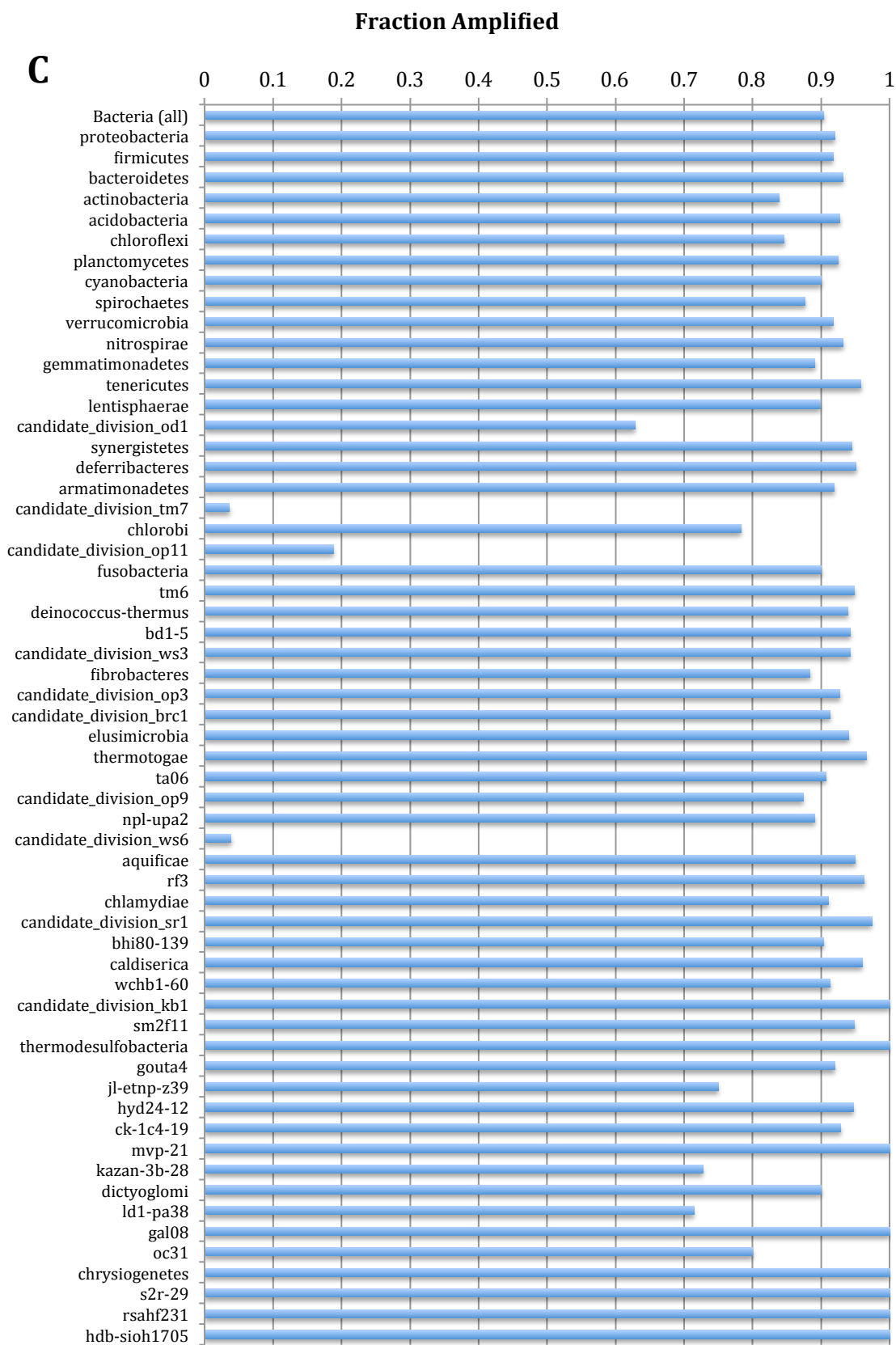

**D**

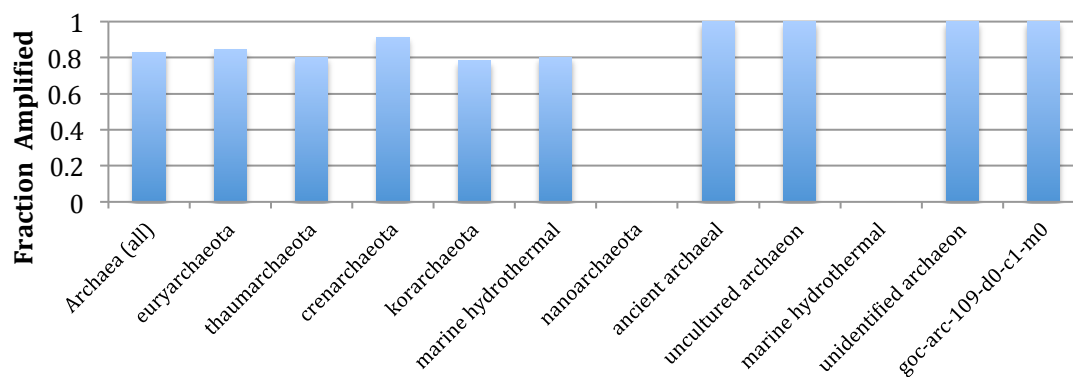

**E**

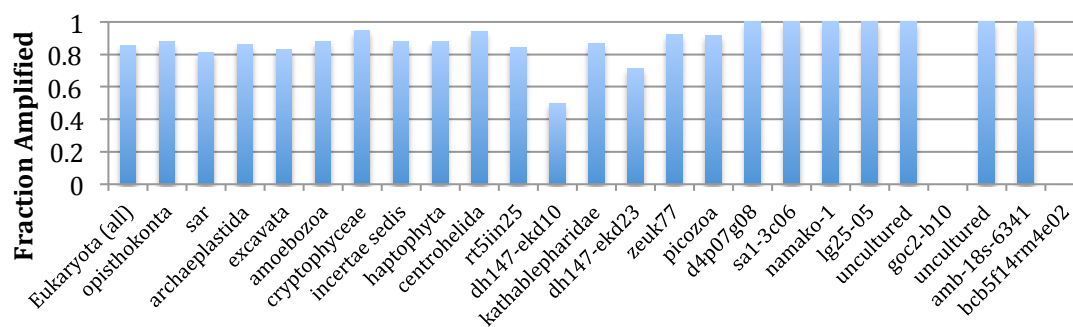

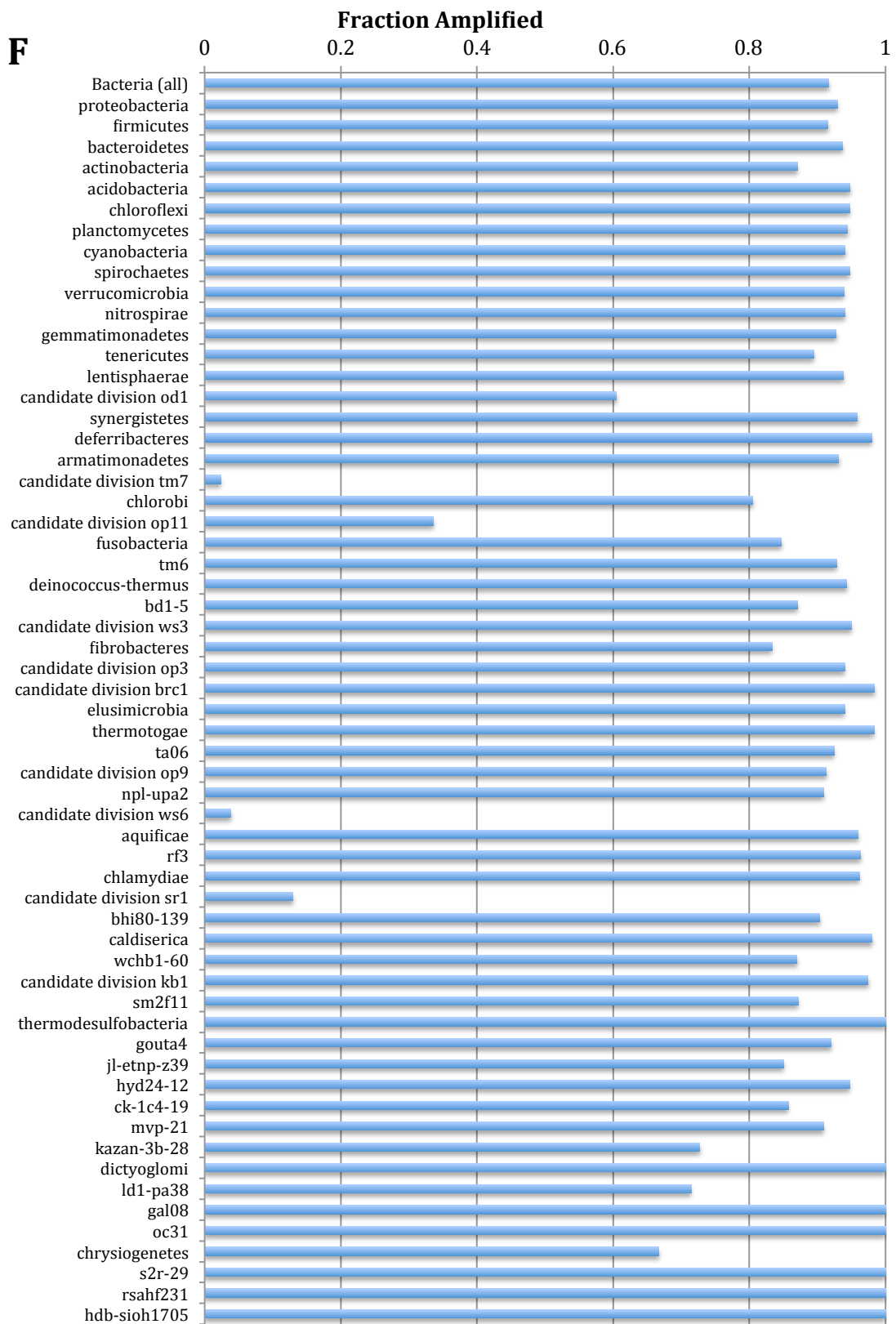

Supplement: Figure S1 [file sys001160029sf5.pdf]
