# Supplementary material for: Improved Bacterial 16S rRNA Gene (V4 and V4-5) and Fungal Internal Transcribed Spacer Marker Gene Primers for Microbial Community Surveys
Source: mSystems. 2015 Dec 22;1(1):e00009-15. doi: 10.1128/mSystems.00009-15 (PMC5069754; doi:10.1128/mSystems.00009-15)

Phyla, AG Fecal

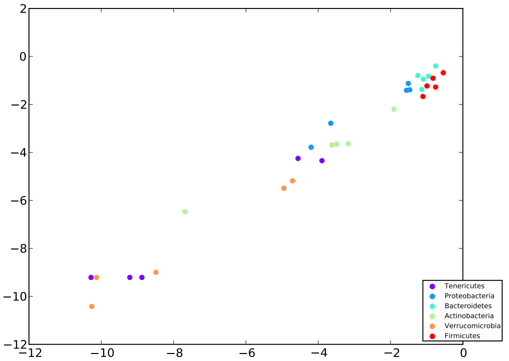

Phyla, AG Skin

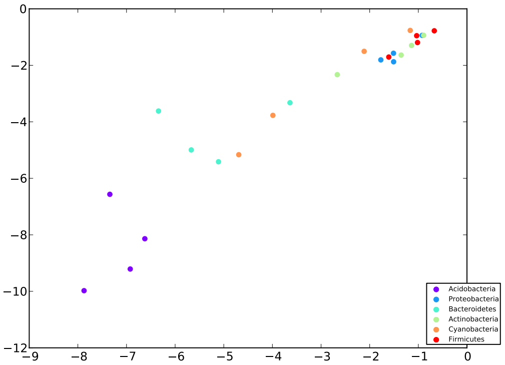

Phyla, Agricultural Soils

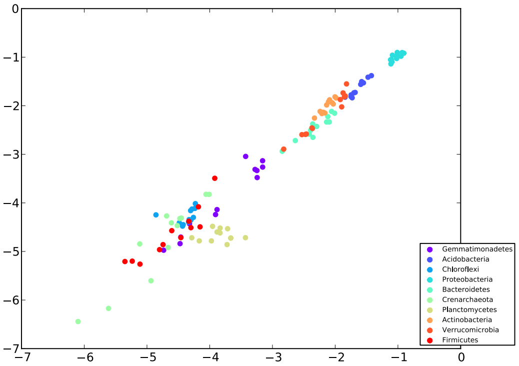

Phyla, EMP Rice Rhizome

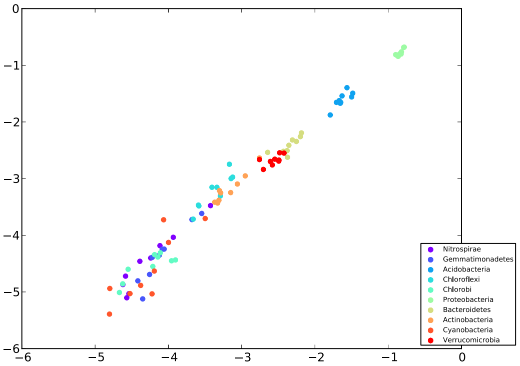

Phyla, Body Farm 1

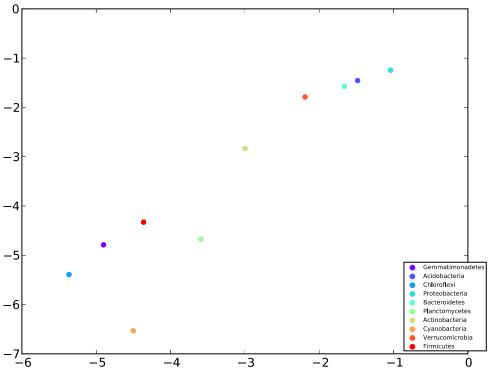

Phyla, Body Farm 2

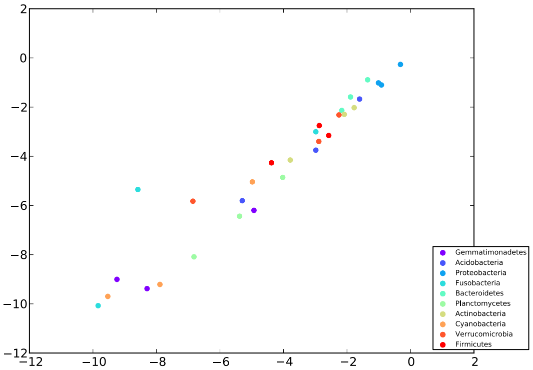

Phyla, Mouse Decomposition

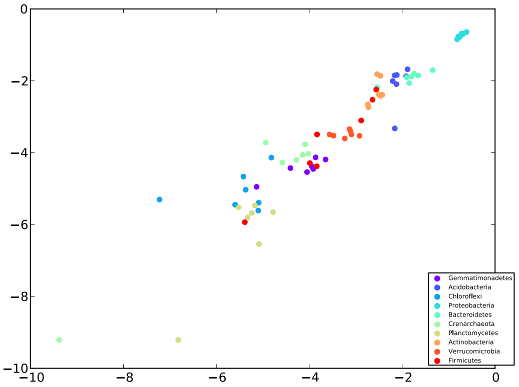

Phyla, Sloan Built Environment

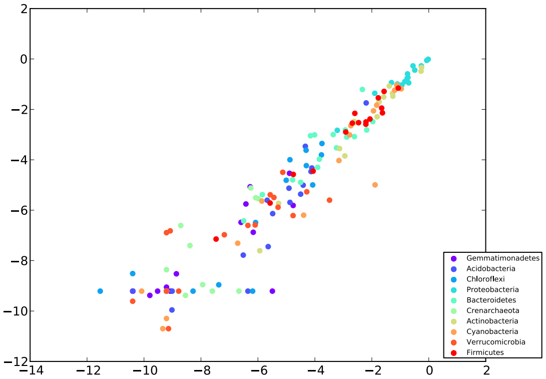

Class, AG Fecal

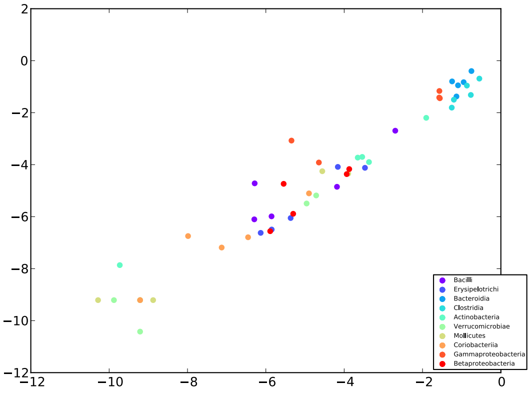

Class, AG Skin

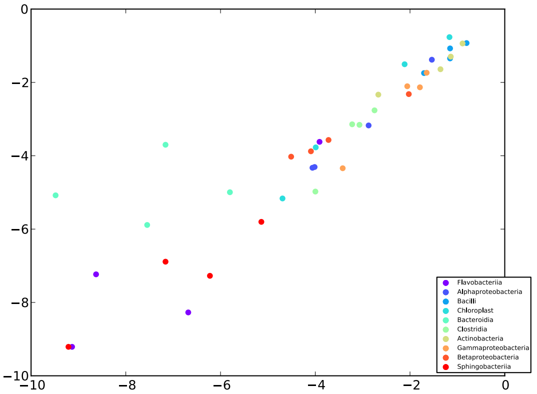

Class, Agricultural Soils

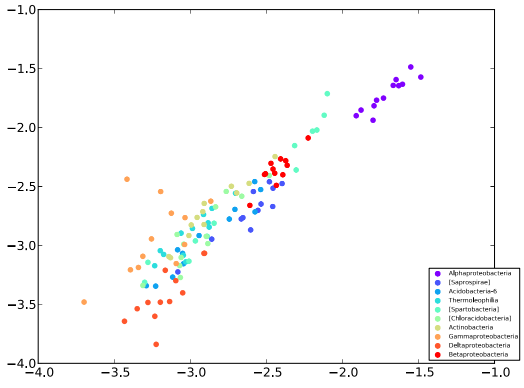

Class, EMP Rice Rhizome

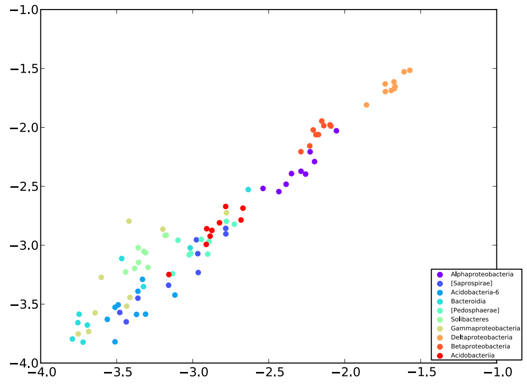

Class, Body Farm 1

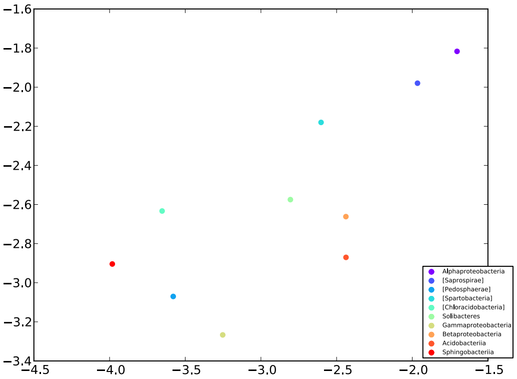

Class, Body Farm 2

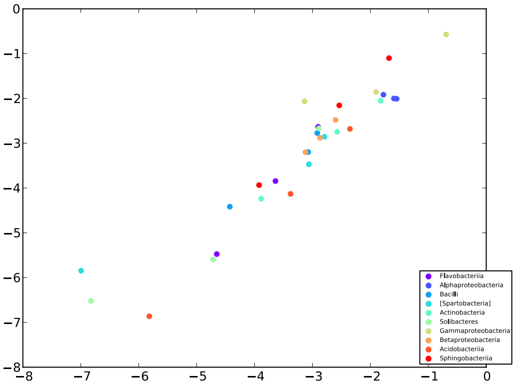

Class, Mouse Decomposition

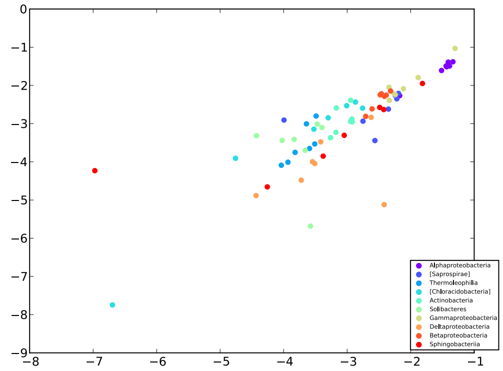

Class, Sloan Built Environment

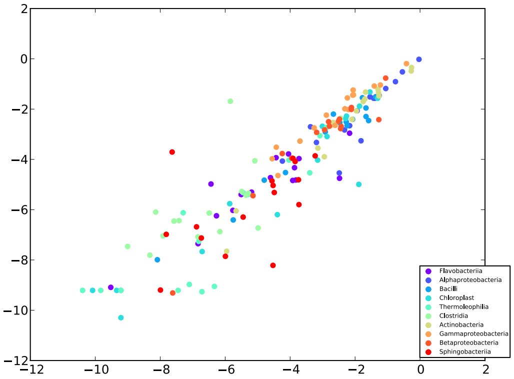

Order, AG Fecal

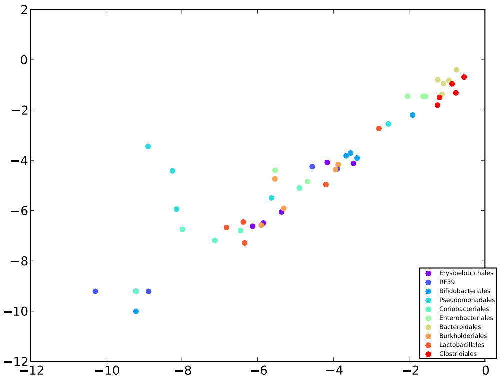

Order, AG Skin

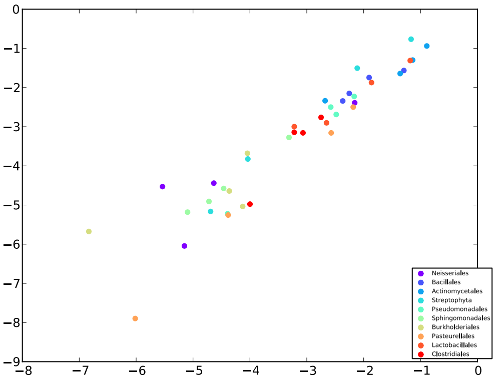

Order, Agricultural Soils

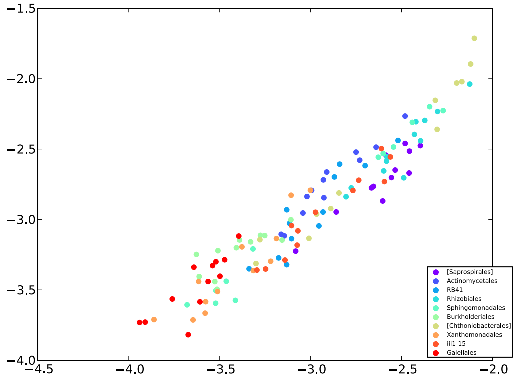

Order, EMP Rice Rhizome

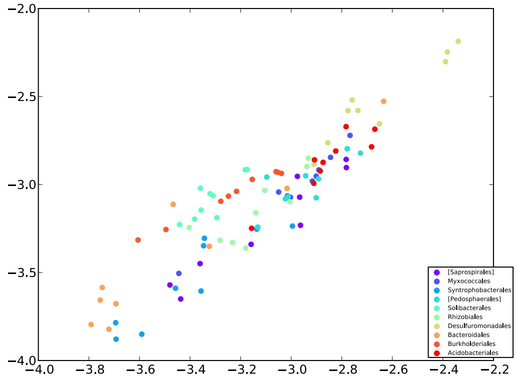

Order, Body Farm 1

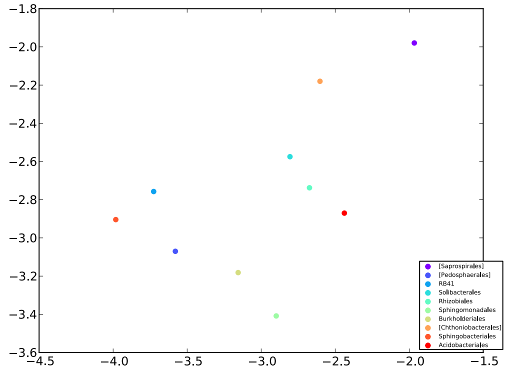

Order, Body Farm 2

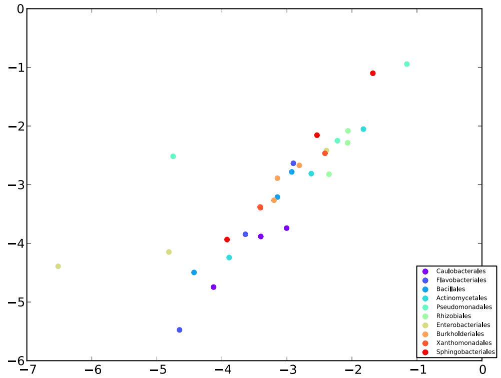

Order, Mouse Decomposition

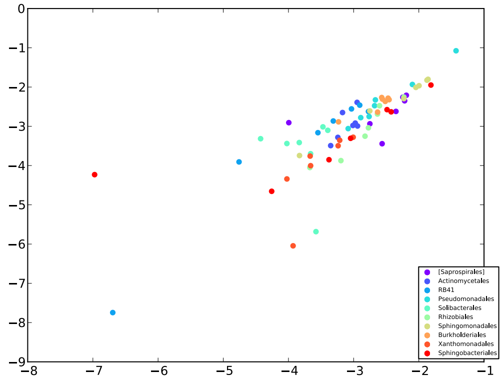

Order, Sloan Built Environment

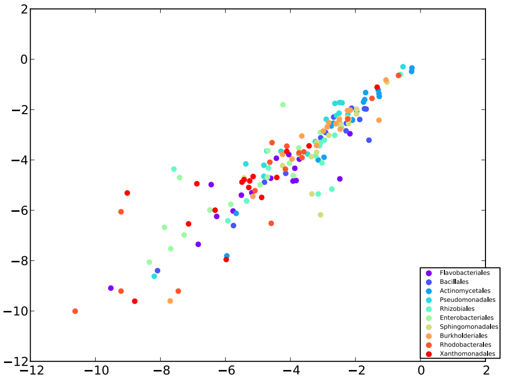

Family, AG Fecal

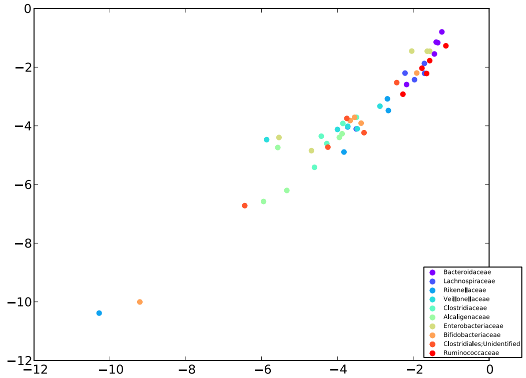

Family, AG Skin

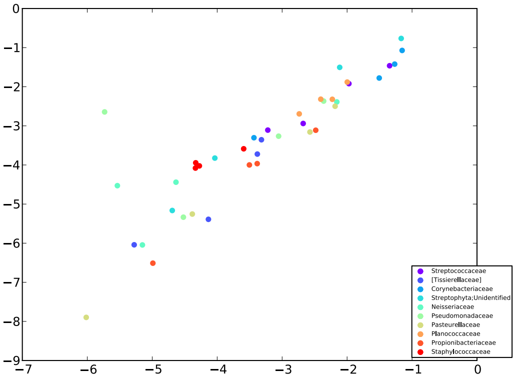

Family, Agricultural Soils

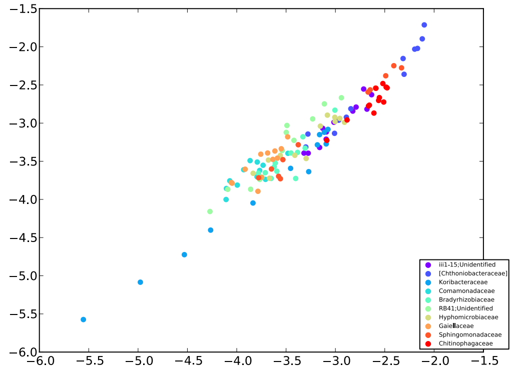

Family, EMP Rice Rhizome

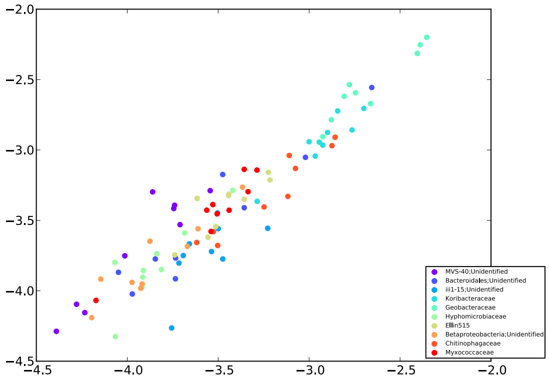

Family, Body Farm 1

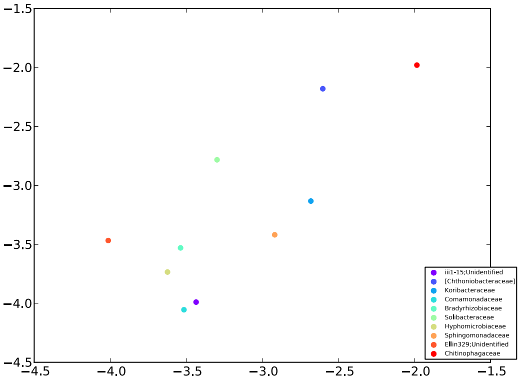

Family, Body Farm 2

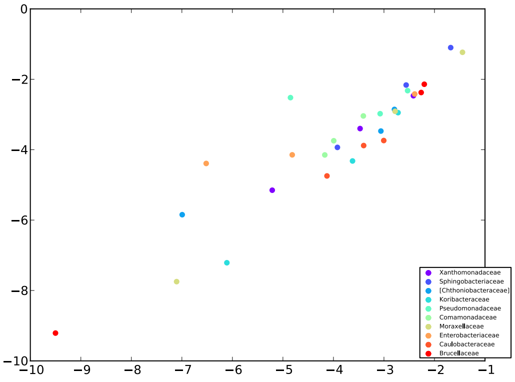

Family, Mouse Decomposition

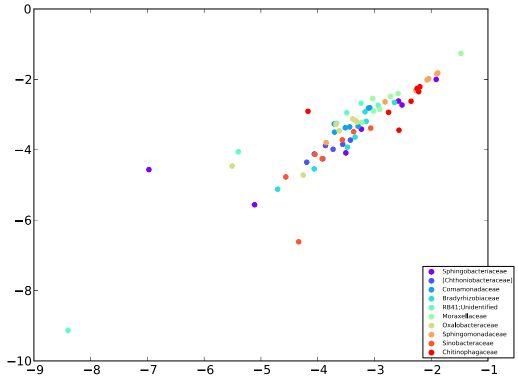

Family, Sloan Built Environment

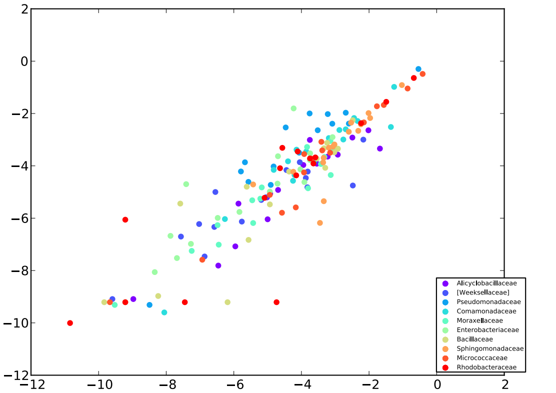

Genus, AG Fecal

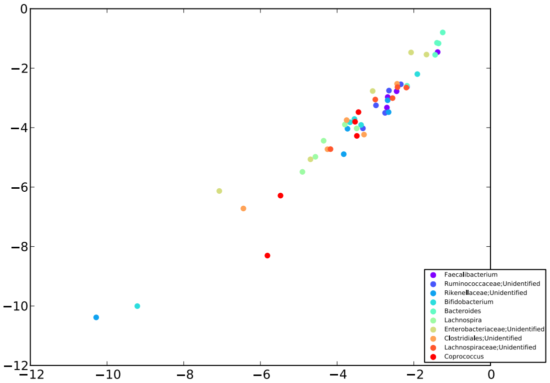

Genus, AG Skin

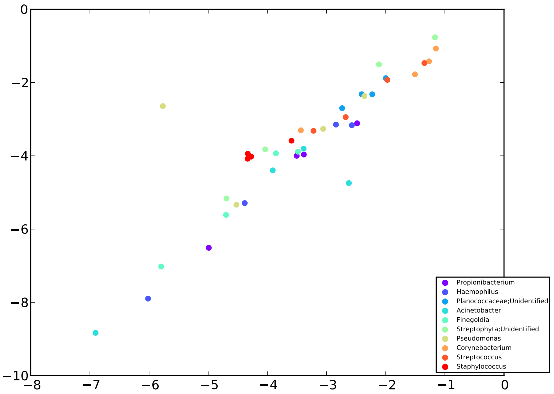

Genus, Agricultural Soils

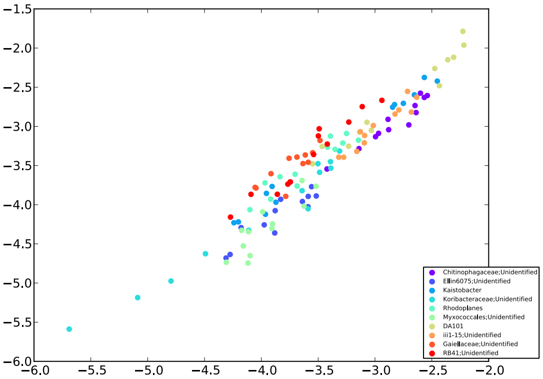

Genus, EMP Rice Rhizome

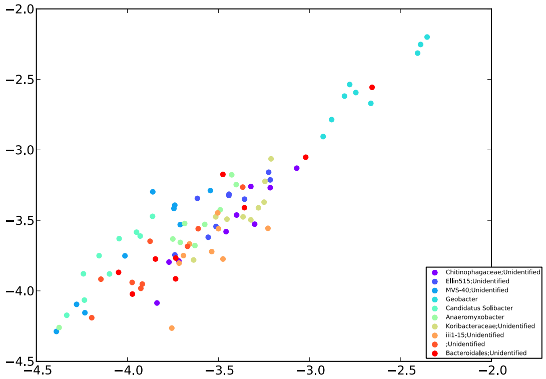

Genus, Body Farm 1

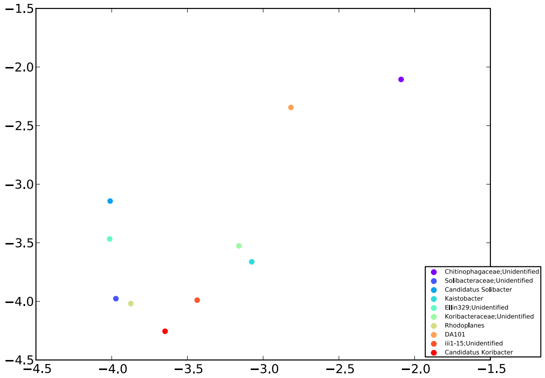

Genus, Body Farm 2

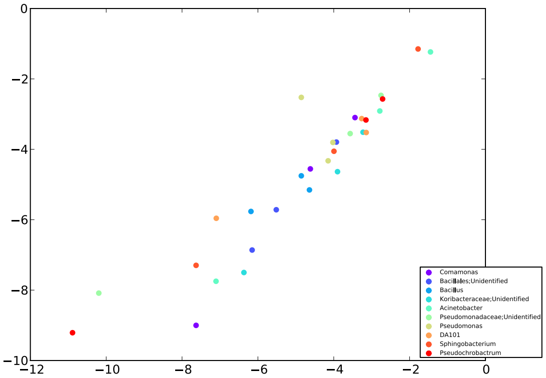

Genus, Mouse Decomposition

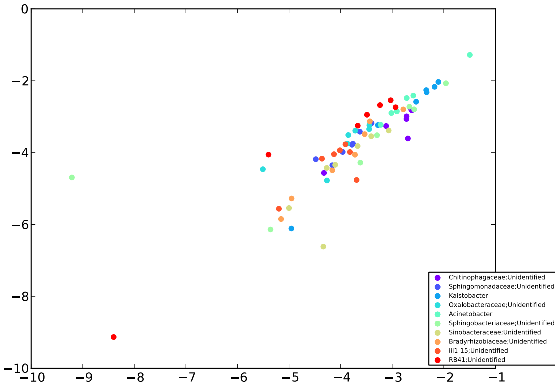

Genus, Sloan Built Environment

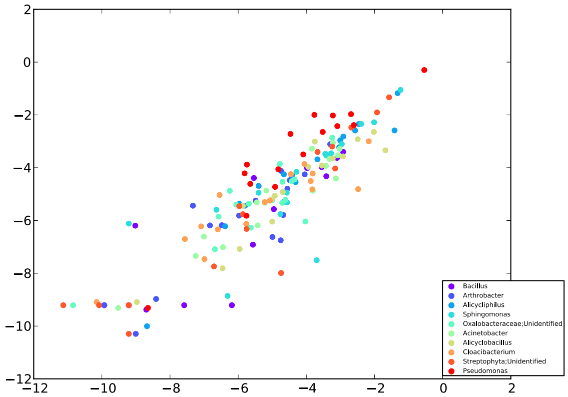

Supplement: Figure S2 [file sys001160029sf6.pdf]
