# Supplementary material for: Improved Bacterial 16S rRNA Gene (V4 and V4-5) and Fungal Internal Transcribed Spacer Marker Gene Primers for Microbial Community Surveys
Source: mSystems. 2015 Dec 22;1(1):e00009-15. doi: 10.1128/mSystems.00009-15 (PMC5069754; doi:10.1128/mSystems.00009-15)
Supplement: Table S2 [file sys001160029st2.docx]

|  | AG fecal | AG skin | Agricultural Soils | Rice rhizome | Body farm 1 | Body farm 2 | Mouse decomp | Sloan built environment (house) |
| --- | --- | --- | --- | --- | --- | --- | --- | --- |
| Phylum | 0.9780 | 0.8833 | 0.9546 | 0.9799 | 0.7508 | 0.9172 | 0.6768 | 0.9148 |
| Class | 0.9434 | 0.8283 | 0.8613 | 0.9398 | 0.2537 | 0.8982 | 0.3406 | 0.8532 |
| Order | 0.8414 | 0.8928 | 0.9178 | 0.8291 | 0.3405 | 0.6644 | 0.3363 | 0.8148 |
| Family | 0.9392 | 0.7712 | 0.9270 | 0.8942 | 0.4144 | 0.8454 | 0.2942 | 0.8161 |
| Genus | 0.9400 | 0.7914 | 0.9082 | 0.8466 | 0.4400 | 0.8690 | 0.3503 | 0.8033 |

**Supplementary Table 2.** Relationship between the original and modified 515f/806r primer pair taxonomy abundances. The R^2^ values for each taxonomic level for each sample type/study (outliers included) are listed.
