# Supplementary material for: Improved Bacterial 16S rRNA Gene (V4 and V4-5) and Fungal Internal Transcribed Spacer Marker Gene Primers for Microbial Community Surveys
Source: mSystems. 2015 Dec 22;1(1):e00009-15. doi: 10.1128/mSystems.00009-15 (PMC5069754; doi:10.1128/mSystems.00009-15)
Supplement: Table S3 [file sys001160029st3.docx]

|  | AG fecal | AG skin | Agricultural Soils | Rice rhizome | Body farm 1 | Body farm 2 | Mouse decomp | Sloan built environment (house) |
| --- | --- | --- | --- | --- | --- | --- | --- | --- |
| Phylum | 0.9733 | 0.6540 | 0.9743 | 0.9156 | 0.1947 | 0.6763 | 0.9232 | 0.8315 |
| Class | 0.9543 | 0.5998 | 0.9398 | 0.7284 | 0.3559 | 0.9643 | 0.9155 | 0.7597 |
| Order | 0.9653 | 0.5027 | 0.9005 | 0.2997 | 0.4590 | 0.9578 | 0.9124 | 0.7949 |
| Family | 0.9528 | 0.6028 | 0.8808 | 0.4555 | 0.2063 | 0.9417 | 0.7478 | 0.7919 |
| Genus | 0.8841 | 0.6525 | 0.8476 | 0.3569 | 0.2637 | 0.9271 | 0.6458 | 0.8084 |

**Supplementary Table 3.** Relationship between the modified 515f/806r and 515f/926r primer pair taxonomy abundances. The R^2^ values for each taxonomic level for each sample type/study (outliers included) are listed.
