# Supplementary material for: Improved Bacterial 16S rRNA Gene (V4 and V4-5) and Fungal Internal Transcribed Spacer Marker Gene Primers for Microbial Community Surveys
Source: mSystems. 2015 Dec 22;1(1):e00009-15. doi: 10.1128/mSystems.00009-15 (PMC5069754; doi:10.1128/mSystems.00009-15)
Supplement: Table S4 [file sys001160029st4.docx]

| **Study** | **Sample IDs** |
| --- | --- |
| American Gut fecal | 1193, 2818, 2036, 1892, 5571 |
| American Gut skin | 5361, 5289, 5081, 5570, 4206 |
| Body farm (human decomposition) 1 (March) | 3.6.13CtrlC, 3.7.13.A, 3.8.13.B, 3.9.13CtrlB |
| Body farm (human decomposition) 2 (May) | 5.11.13.C.CtrlA, 5.11.13.S.RA.G, 5.13.13.S.LA.F, 5.9.13.S.CtrlB, 5.9.13.S.H.A |
| Mouse decomposition control soil | D.soil.T0.1, D.soil.T0.2, D.soil.T0.3, D.soil.T0.4, D.soil.T0.5, F.CTRL.soil.T0.1, F.CTRL.soil.T0.2, F.CTRL.soil.T0.3, F.CTRL.soil.T0.4, F.CTRL.soil.T0.5, G.CTRL.soil.T0.1, G.CTRL.soil.T02, G.CTRL.soil.T0.3, G.CTRL.soil.T0.4, G.CTRL.soil.T0.5 |
| Sloan Toronto house | DM51, DM150, DM3, AG34, AG7, AG10, JB.lh, JB3, DM99, DM74, RK15, DM97, DM105, AG45, DM145, DM186, DM152, RK46, DM187, AG35, BVP6, AG27, AG72, DM147, DM111, DM95, RK9, RK49, DM117, RK35, AG3.ls, DM73 |
| Agricultural soils | T1-R3-6, T7-R1-6, T7-R4-6, T7-R6-6, T1-R6-6, T7-R5-6, T1-R2-6, T1-R1-6, T7-R2-6, T1-R4-6, T7-R3-6, T1-R5-6 |
| Rice rhizome | MS00024, MS00032, MS00048, MS000117, MS000118, MS000119, MS000238, MS000253, MS000586 |

**Supplementary Table 4.** List of studies and sample IDs for samples amplified with the primer pairs used in this study.
